# Transgenic expression of endoplasmic reticulum proteins in *C. elegans* neurons is prone to causing ER stress

**DOI:** 10.17912/micropub.biology.001547

**Published:** 2025-05-29

**Authors:** Junhyun Park, Shaul Yogev

**Affiliations:** 1 Neuroscience, Yale University, New Haven, Connecticut, United States

## Abstract

In studying the endomembrane system, organelle-specific markers tagged with fluorescent proteins are used to visualize individual organelles. However, whether the expression of organelle marker perturbs the organelle's biology is not always apparent. We report that expression of a GFP-tagged Endoplasmic Reticulum (ER) protein causes low levels of ER stress that are challenging to detect in control animals. This stress is revealed only once the ER-associated degradation (ERAD) pathway is compromised. Our results highlight the vulnerability of the ER and suggest that the possible contribution of ER stress to phenotypes obtained with transgenic markers should be considered when interpreting the phenotypes.

**Figure 1. Transgenic expression of endoplasmic reticulum proteins in C. elegans neurons is prone to causing ER stress:**
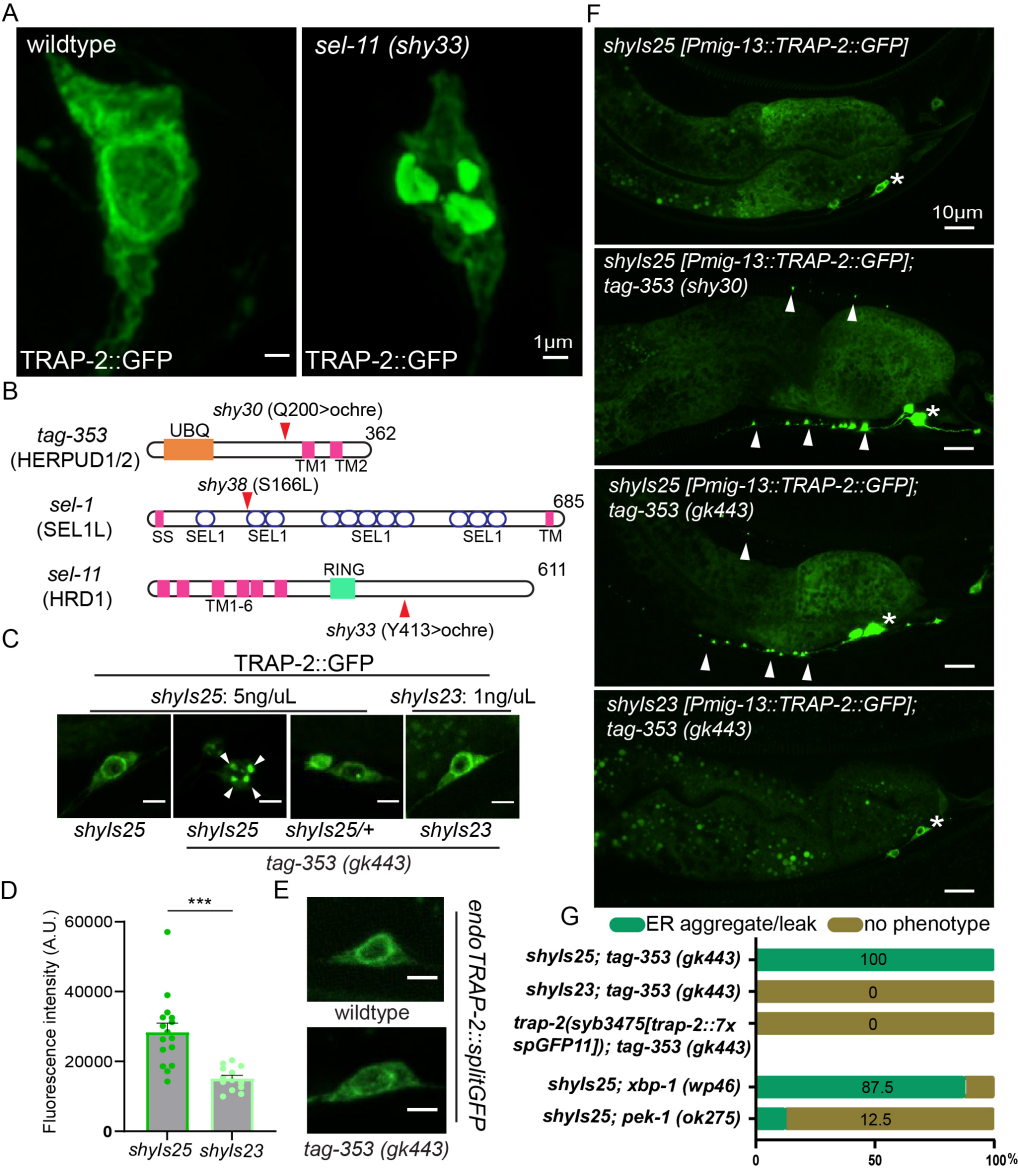
(A) Representative images of AWC neuronal soma expressing TRAP-2 ::GFP transgene ( *shyIs17* ) in wildtype and *sel-11 (shy33)* mutant. Scale bar= 1µm. (B) Diagram of mutations isolated from a mutagenic screen using TRAP-2 ::GFP transgene expressing animals. (C) Representative images of ER protein aggregates found in *tag-353* (HERPUD1/2 ortholog) null mutant ( *gk443* ) with different level of TRAP-2 ::GFP expression. Scale bar= 3µm. (D) Quantification of TRAP-2 ::GFP fluorescence intensity in DA9 neuronal soma of two different transgenes *shyIs25* and *shyIs23* . n=13-16. ^∗∗∗^ p < 0.0001 (two-tailed t-test). (E) Representative images of endogenous TRAP-2 labeled with splitGFP in wildtype and in *tag-353* null mutant ( *gk443* ). Scale bar= 3µm. (F) TRAP-2 ::GFP localization in DA9/VA12 neurons in wildtype, *shy30* , and *gk443* mutant. White asterisk indicates the neuronal soma. White arrow heads show TRAP-2 ::GFP proteins leaking into neurites. Scale bar= 10µm. (G) Percent penetrance of ER aggregate leakage phenotype in different mutants. n=8-10.

## Description

To investigate genes required for neuronal endoplasmic reticulum (ER) homeostasis, we generated *C. elegans* strains stably expressing a GFP-tagged rough endoplasmic reticulum protein, TRAP-2 , in either AWB/AWC ( *shyIs17* ) or DA9 neurons ( *shyIs25* , Figure 1A and 1F top panel). In both strains, TRAP-2 displayed the expected localization restricted to neuronal soma where rough ER proteins were natively concentrated ( Figure 1F top panel; Lindsey and Ellisman, 1985; Rolls et al., 2002) and was enriched in the peri-nuclear region ( Figure 1A and 1C). A similar pattern was observed when TRAP-2 was tagged at the endogenous locus with seven repeats of splitGFP ( Figure 1E ). We did not detect any ER morphology defects or potential artifacts caused by multimerization of GFP in these transgenic animals ( Figure 1A and 1C left; Snapp et al., 2003). Thus, overexpressed TRAP-2 ::GFP was “well behaved” by the criteria that are routinely used to evaluate organelle markers in *C. elegans* .

From a forward visual genetic screen using these strains, we isolated multiple mutants with clear defects in ER morphology and with TRAP-2 ::GFP aggregates leaking into both dendrites and axons ( Figure 1A right and 1F). Using whole genome sequencing and transgene rescue, we mapped three of these mutations from three separate mutants to *tag-353* (herp or HERPUD1/2 in mammals), *sel-1* (SEL1L in mammals), and *sel-11* (HRD1 in mammal) - genes known to be involved in ER stress and Unfolded Protein Response (UPR; Hwang and Qi, 2018).

*tag-353* ( *shy30)* and *sel-11* ( *shy33)* mutations resulted in early stop codons where for *tag-353* ( *shy30)* , the ochre mutation truncated the protein before its transmembrane domain ( Figure 1B ). *shy33* mutation spared all six transmembrane domains and luminal RING domain of the E3 ubiquitin ligase but it lost highly conserved disordered region that is important for stabilizing HRD1-SEL1L-HERP complex ( Figure 1B *;* Schulz et al., 2017). *shy38* mutant carried a point mutation prior to the second sel-1-like repeat ( Figure 1B ). The ER defect phenotype was 100% penetrant for *shy30* and *shy33* and roughly 50% penetrant for *shy38* ( Figure 1G ).

The isolation of several ER-stress genes in our screen raised the concern that the observed phenotypes were due to ER-stress induced by the transgenic expression of TRAP-2 ::GFP, and do not necessarily reflect the biology of endogenous TRAP-2 . In agreement with this interpretation, HERPUD1 has been reported to become active after ER stress has been induced (Kokame et al., 2000; Leitman et al., 2014), and studies in mammalian NIH 3T3 cells suggest that transient overexpression of ER proteins can cause UPR (Kamhi-Nesher et al., 2001; Kondratyev et al., 2007). To test if TRAP-2 ::GFP expression level was responsible for the ER phenotypes, we examined *tag-353 ( gk443 )* null mutants that were either homozygous or heterozygous for the TRAP-2 ::GFP transgene *shyIs25* . We found that reducing transgene expression either eliminated or reduced the ER phenotypes in most animals ( Figure 1C ). To corroborate this finding, we used an independent transgene ( *shyIs23* ) that was generated with a lower concentration of the TRAP-2 ::GFP plasmid (1 ng/ml) compared to *shyIs25* (5 ng/ml) and was ~50% fainter ( Figure 1D ). Indeed, we did not detect ER phenotypes in *tag-353* ( *gk443* ) mutants with this transgene. Lastly, we tagged endogenous *trap-2* with seven repeats of splitGFP and again could not detect ER phenotypes with the null ERAD mutant tested ( Figure 1E and 1G). Although the brightness of splitGFP on endogenous TRAP-2 may vary depending on the expression level of splitGFP components, with *shyIs32* transgene stably expressing GFP1-10 in DA9, we find that, qualitatively, endogenous TRAP-2 had similar brightness to that of *shyIs23* transgene and would recommend using it over an extrachromosomal array (Zhang et al., 2024). We interpret these results to suggest that above a certain threshold, overexpression of ER proteins, while not showing a visible morphological defect in control animals, may raise basal levels of ER stress. This effect is then manifest when the machinery that deals with ER stress is compromised, as in our mutants.

To further test if ER stress response was indeed suppressing TRAP-2 ::GFP aggregate formation in our control transgene *shyIs25* ( Figure 1F top panel), we crossed it to mutants for ER stress upstream genes: *xbp-1* and *pek-1* . Consistent with previous reports in *C. elegans* that HERPUD1/2 is active under the *xbp-* 1 branch of ER stress, *xbp-1* null mutants phenocopied the *tag-353* mutants while *pek-1* mutants did not ( Figure 1G, Shen et al., 2005). Taken together, our results suggest that despite the fact that TRAP-2 ::GFP appears similar in control animals whether it is highly expressed, mildly expressed, or endogenously tagged, the higher expression transgene triggers an *xbp-1* and *tag-353* dependent ER stress response that is preventing aggregation.

Fluorescence reporter strains are commonly used for genetic screens and assays in *C. elegans* . Often, endogenously-tagged proteins yield a fluorescent signal that is too low for a visual genetic screen, necessitating the use of transgenes. However, the cellular stress that these reporters may cause is not always addressed if the reporter appears correctly localized. Here, we share a cautionary example where expression of a GFP-tagged ER protein caused ER stress that was not visibly detectable by the distribution of the protein in control animals. Since this reporter was expressed in a small number of cells within an animal, it is doubtful whether methods such as Western Blotting or RT-PCR would have detected this stress. Beyond highlighting the sensitivity of the ER to protein overexpression, our results emphasize the importance of using orthogonal validation methods when inferring protein and organelle distribution with fluorescent markers.

## Methods

**Strains and maintenance**

All *C. elegans* strains were grown on nematode growth medium plates seeded with *E. coli* OP50 . The N2 Bristol strain was used as wildtype and for outcrosses. All animals were grown at 20°C for experiments.

**Transgenic *C. elegans* strain generation**

Transgenic animals were generated by injecting plasmids into the gonads of young adult animals following standard protocols. Resulting F1s transmitting the injected expression arrays to its progenies were singled. Animals with preferred transmission rate were mounted on slide glass and were visually screened for desired expression level of extrachromosomal array on an inverted microscope. For stable expression of the array, animals carrying desired extrachromosomal arrays were incubated in 30ug/ml TMP dissolved M9 media and subsequently irradiated with ultraviolet light at 300 microjoules/cm ^2^ × 100. Animals that have stably integrated the array were then selected and outcrossed at least 4 times to rid background mutations. CRISPR Knock-in of 7XGFP11 to the endogenous *trap-2* locus ( *trap-2* ( *syb3475* )) was generated by SunyBiotech.

**Forward genetic screen**

We performed a visual forward F2 genetic screen surveying∼ 2,000 haploid genomes. Ethyl methanesulfonate (EMS) was used to induce random germline mutations in animals carrying the *shyIs25* [ *Pmig-13:: trap-2 ::GFP, Podr-1::RFP* ] or *shyIs17* [ *Podr-1:: trap-2 ::GFP, Punc-122::RFP* ] markers. Mutagenized worms were scored for changes in TRAP-2 ::GFP localization in DA9 or AWB/AWC neurons. Homozygous mutants were rescued and outcrossed 5 times with N2 males prior to phenotypic analysis and whole-genome sequencing (Balseiro-Gómez, Park et al., 2022)

**Fluorescence microscopy and sample preparation**

L4 larvae animals were grown at 20°C, 24 hours prior to imaging. One day adult animals were mainly used for quantification and assessment of phenotype as previously described (Park et al., 2023). Andor Dragonfly spinning-disk confocal microscope equipped with a plan apochromat objective (63x, 1.4 NA, oil) and a Zyla scientific CMOS camera was used for standard fluorescence imaging. For high resolution imaging, Airyscan function of Carl Zeiss LSM880 confocal laser scanning microscope (63x, 1.4 NA, plan-apochromat, oil) with Airyscan detector was used. Raw Airyscan images were processed using ZEN imaging software (Zeiss). Raw imaging files were imported to ImageJ for quantification of fluorescence intensity. ImageJ version 1.49 (NIH) was used for all image processing and intensity quantification.

**Statistical analysis**

Statistical analysis was performed on Prism 9 (GraphPad) and Microsoft Excel. Data were considered significant at *p* ≤ 0.050 with statistical test indicated in figure legend.

## Reagents

**Table:** 

**Strain name**	**Genotype**	**Source**
MTS349	* shyIs17 [Podr-1:: trap-2 ::GFP, Punc-122::rfp] *	This Study
MTS395	* shyIs17 [Podr-1:: trap-2 ::GFP, Punc-122::rfp]; shy33 *	This Study
MTS387	* shyIs25 [Pmig-13:: trap-2 ::GFP, Podr-1::rfp] *	This Study
MTS392	* shyIs25 [Pmig-13:: trap-2 ::GFP, Podr-1::rfp]; shy30 *	This Study
MTS612	* shyIs25 [Pmig-13:: trap-2 ::GFP, Podr-1::rfp]; shy30; shyEx161 [WRM0635aE10; Podr-1::gfp] (fosmid) *	This Study
MTS508	* shyIs25 [Pmig-13:: trap-2 ::GFP, Podr-1::rfp]; shy30; shyEx113 [Pmig-13:: tag-353 ::SL2::mCh; Podr-1::GFP] *	This Study
MTS570	* shyIs25 [Pmig-13:: trap-2 ::GFP, Podr-1::rfp]; cup-2 & tag-353 ( gk443 ) *	*cup-2 & tag-353 ( gk442 )* is from CGC
MTS422	* shyIs25 [Pmig-13:: trap-2 ::GFP, Podr-1::rfp]; shy38 *	This Study
MTS610	* shyIs25 [Pmig-13:: trap-2 ::GFP, Podr-1::rfp]; shy38; shyEx160 [Pmig-13:: sel-1 ::SL2::mCh; Podr-1:gfp] *	This Study
MTS615	* shyIs25 [Pmig-13:: trap-2 ::GFP, Podr-1::rfp]/+; cup-2 & tag-353 ( gk443 ) *	This Study
MTS376	* shyIs23 [Pmig-13:: trap-2 ::GFP, Podr-1::rfp] *	This Study
MTS571	* shyIs23 [Pmig-13:: trap-2 ::GFP, Podr-1::rfp]; cup-2 & tag-353 ( gk443 ) *	This Study
PHX3475	*trap-2 (syb3475 [trap-2::GFP11x7]* )	SunyBiotech
MTS893	*trap-2 (syb3475 [trap-2::GFP11x7]); shyIs32 [Pmig-13::gfp1-10, Podr-1::rfp] *	This Study
MTS2255	*trap-2 (syb3475 [trap-2::GFP11x7]); shyIs32 [Pmig-13::gfp1-10, Podr-1::rfp]; cup-2 & tag-353 ( gk443 ) *	This Study
MTS569	* shyIs25 [Pmig-13:: trap-2 ::GFP, Podr-1::rfp]; xbp-1 ( wp46 ) *	*xbp-1* ( *wp46* ) is from Hammarlund lab
MTS572	* shyIs25 [Pmig-13:: trap-2 ::GFP, Podr-1::rfp]; pek-1 ( ok275 ) *	*pek-1 ( ok275 )* is from CGC
